# Evaluation of Oscillatory Flow Conditions for Microalgal CO_2_ Capture and Biomass Sedimentation Kinetics: Experimental and Mathematical Approach

**DOI:** 10.3390/biotech15020036

**Published:** 2026-05-23

**Authors:** Inês S. Almeida, Eva M. Salgado, António M. A. Ferreira, José C. M. Pires

**Affiliations:** LEPABE, ALiCE, Faculty of Engineering, University of Porto, Rua Dr. Roberto Frias, 4200-465 Porto, Portugal; ines.almeida@tu-dresden.de (I.S.A.); antonio@fe.up.pt (A.M.A.F.)

**Keywords:** *Chlorella vulgaris*, CO_2_ biofixation, hydrodynamic evaluation, microalgae cultivation systems, nutrient removal, process modelling

## Abstract

This study evaluates the oscillatory frequency and amplitude in an oscillatory flow reactor with smooth periodic constrictions (OFR-SPC) for the cultivation and harvesting of *Chlorella vulgaris* fed with an air stream with 5% (*v*/*v*) CO_2_. Their effect on biomass productivity, CO_2_ capture, nutrient removal, and sedimentation kinetics was assessed. Cultures were tested at frequencies of 0.5–2.5 Hz and amplitudes of 6–18 mm. At 2.5 Hz|6 mm, the system achieved the maximum biomass concentration (592 mg_DW_ L^−1^), productivity (5.36 mg_DW_ L^−1^ h^−1^), and CO_2_ fixation (8.34 mg L^−1^ h^−1^) as well as complete nitrogen removal and near-complete phosphorus removal (100% and 91%, respectively). Complete sedimentation occurred at 0.5 Hz|6 mm, with kinetics described by the Gompertz model (k = 4.60 h^−1^), confirming the feasibility of low-cost biomass recovery. Additionally, zeta potential positively influenced sedimentation but negatively affected productivity. Statistical analyses confirmed oscillation frequency and amplitude as key factors, establishing the OFR-SPC as a promising technology for microalgae-based efficient CO_2_ capture, nutrient removal, and low-cost biomass harvesting.

## 1. Introduction

The era of the industrial technology revolution, alongside widespread reliance on fossil fuels, has led to a gradual increase, approximately 75%, in greenhouse gas (GHG) emissions over the past two centuries [1]. Substantial investments in CO_2_ capture are anticipated to play a vital role in reducing emissions, particularly from power plants. In this context, employing biological techniques utilising microorganisms to capture and convert CO_2_ into food, chemicals, or fuels offers a natural, renewable, and sustainable approach that enables biomass utilisation and co-benefits for wastewater treatment [2]. Among the array of potential systems for capturing CO_2_, microalgae stand out. These microorganisms, characterised by their photosynthetic nature, utilise CO_2_ and sunlight to produce O_2_ and valuable by-products, such as biofuels [3]. Microalgae play a significant role in the carbon cycle, accounting for 50% of the total fixation of atmospheric CO_2_, making them a promising technology for the biological capture of CO_2_ [4]. In contrast to terrestrial plants, microalgae show higher growth rates, biomass productivity, and CO_2_ fixation efficiencies that are between 10 and 50 times higher [5]. Furthermore, microalgae can be grown on non-fertile land and utilise nutrients from different wastewater sources, which enhances sustainability [6]. Adopting a microalgae-based biological approach to carbon capture offers a promising avenue for mitigating carbon footprints and generating bioenergy, presenting a CO_2_-neutral alternative to fossil fuels. Despite the inherent advantages of microalgae-based methodologies, parameters such as CO_2_ mass transfer and biomass harvesting remain constraints that inhibit the process from becoming widespread and its utilisation in the production of high-value compounds [1]. CO_2_’s low solubility in the liquid medium leads to concentration gradients between the gas and liquid phases, which are influenced by factors such as temperature, pressure, and solvent properties. Without a gradient between the gas and liquid phase concentrations, mass transfer does not occur [6]. On the other hand, biomass harvesting, which occurs after biomass growth, involves removing the microalgae from the suspension culture and is a crucial step in microalgae production. The major challenge at this stage is that harvesting accounts for approximately 20–30% of the total cost of biomass production due to the small cell size (<30 µm) and low concentration and dilution nature of the microalgae growth in the culture medium (<1 g L^−1^) [7]. These limitations highlight the urgent need for cultivation systems that simultaneously enhance CO_2_ mass transfer and biomass productivity while facilitating low-cost harvesting via passive sedimentation.

Several microalgae cultivation systems have been tested for carbon fixation. Beigbeder et al. [8] demonstrated the potential of an integrated microalgae system for capturing CO_2_ and generating bioproducts. Firstly, *Parachlorella kessleri* was cultivated in NaHCO_3_-based media with pH control to enhance inorganic carbon (IC) removal and microalgae growth. Then, a two-step cultivation strategy was designed to couple CO_2_ capture from fermentation with microalgae cultivation in bubble column photobioreactors (PBRs), though carbon utilisation remained limited by CO_2_ loss during agitation. Nad’ et al. [9] developed and tested a new vertical PBR with elliptical tubes using the microalga *Chlorella pyrenoidosa*, achieving CO_2_ biofixation and flue gas pollutant absorption, though biomass productivity declined significantly when scaling from the laboratory to pilot level. Mitra et al. [10] evaluated the addition of bicarbonate as a dissolved inorganic carbon source in bubble column PBRs with *C. vulgaris*, improving carbon fixation and biomass productivity, though the approach remained dependent on external chemical supplementation rather than direct CO_2_ supply. Additionally, *C. vulgaris* exhibited enhanced self-flocculation (55% improvement) in the presence of bicarbonate, a phenomenon supported by the production of extracellular polymeric substances (EPSs) by microalgae, which facilitates biomass harvesting. Rezvani and Farazmand [11] optimised light/dark cycles and N:P ratios for *Scenedesmus* sp. in a bubble column PBR, achieving nitrate removal and CO_2_ fixation, but required hydraulic retention times of 3–4 days and generated elevated effluent COD, limiting the treated water quality. Rezvani [12] further demonstrated that mixed cultures of *Chlorella vulgaris* and *Scenedesmus* sp. at a 2:1 inoculation ratio outperformed the monoculture, reaching a nitrate removal rate of 13.6 mg/L/d and a CO_2_ fixation rate of 0.41 g/L/d, yet increased COD production remained a constraint. Rezvani and Sarrafzadeh [13] subsequently combined microalgae with heterotrophic denitrifies in a sequential batch photobioreactor, improving both the nitrate removal rates and biomass sedimentation through synergistic interactions. However, none of these systems simultaneously addresses biomass productivity, nutrient removal, and sedimentation in microalgae cultures under controlled hydrodynamic conditions.

Gonçalves et al. [14] used an oscillatory flow reactor with smooth periodic constrictions (OFR-SPC) to evaluate CO_2_ mass transfer in *C. vulgaris* cultures. The authors found that this PBR could be an effective option for culturing microalgae, competing with traditional bubble column and airlift reactors. The OFR-SPC uses controlled oscillatory movement to enhance mixing and mass transfer. Thus, the OFR-SPC offers two key advantages [15]: (i) an enhanced gas–liquid mass transfer area through reduced bubble size, and (ii) prolonged bubble residence time, both contributing to improved CO_2_ availability in the culture. While Gonçalves et al. [14] characterised CO_2_ gas–liquid mass transfer in the OFR-SPC to sustain microalgae growth, the present work evaluates, for the first time, the oscillatory parameters—frequency and amplitude—for the simultaneous improvement of *C. vulgaris* growth, nutrient removal, and passive sedimentation as combined objectives, maintaining a fixed CO_2_ input concentration of 5% (*v*/*v*) [16,17]. The specific objectives are to evaluate the effect of oscillatory variables on: (i) *C. vulgaris* growth by measuring its biomass concentration, specific growth rate, and biomass productivity; (ii) nutrient removal from the medium by determining nitrogen and phosphorus concentration over time and corresponding removal rates; and (iii) sedimentation kinetics and efficiency.

## 2. Materials and Methods

### 2.1. Microorganisms and Culture Medium

*C. vulgaris* CCAP 211/11B was acquired from the Culture Collection of Algae and Protozoa (CCAP, Oban, UK). Stock solutions were prepared in 100-mL Erlenmeyer flasks using the modified OECD (Organisation for Economic Cooperation and Development) test medium, with the following composition, per litre: 250 mg NaNO_3_, 12 mg MgCl_2_·6H_2_O, 18 mg CaCl_2_·2H_2_O, 15 mg MgSO_4_·7H_2_O, 45 mg KH_2_PO_4_, 0.08 mg FeCl_3_6H_2_O, 0.1 mg Na_2_EDTA·2H_2_O, 0.185 mg H_3_BO_3_, 0.415 mg MnCl_2_·4H_2_O, 3 µg ZnCl_2_, 1.5 µg CoCl_2_·6H_2_O, 0.01 µg CuCl_2_·2H_2_O, 7 µg Na_2_MoO_4_·2H_2_O, and 50 mg NaHCO_3_ [14]. Erlenmeyer flasks were kept at room temperature with a light-emitting diode (LED) panel providing a light intensity of roughly 50 µmol m^−2^ s^−1^. A Unimax 1010 orbital shaker (Heidolph, Schwabach, Germany) with a speed setting of 120 rotations per minute (rpm) was used to agitate the cultures. Following a 30-day cultivation period, the stock cultures were transferred into 2-L glass bottles exposed to a light intensity of 180 µmol m^−2^ s^−1^. Then, the cells were harvested using a Selecta Centromix S-549 centrifuge (PI El Nogal, Torremolinos, Spain) at room temperature for 10 min to inoculate the OFR-SPC, with an initial biomass concentration of approximately 130 mg_DW_ L^−1^.

### 2.2. Experimental Setup

**Figure 1:**
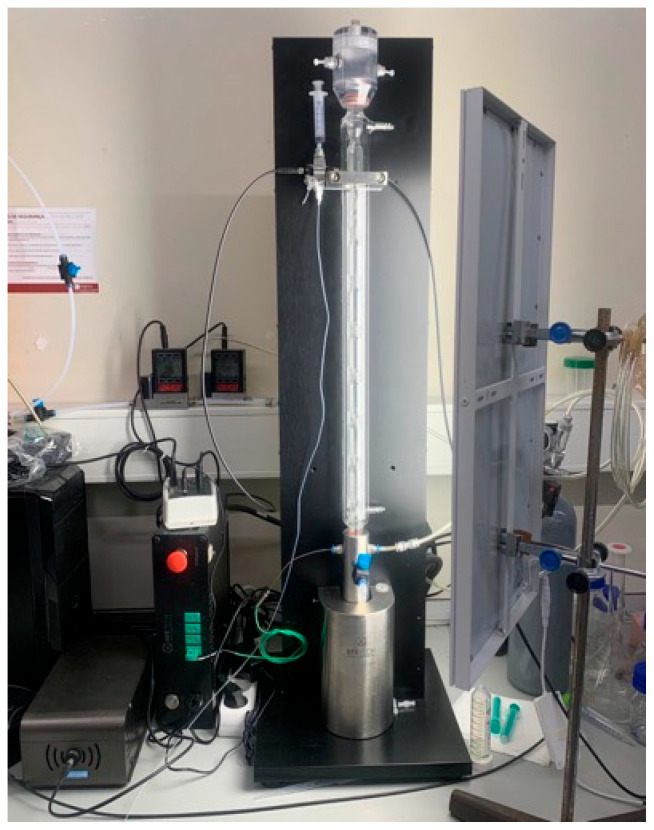
Photograph of the experimental setup.

**Figure A1:**
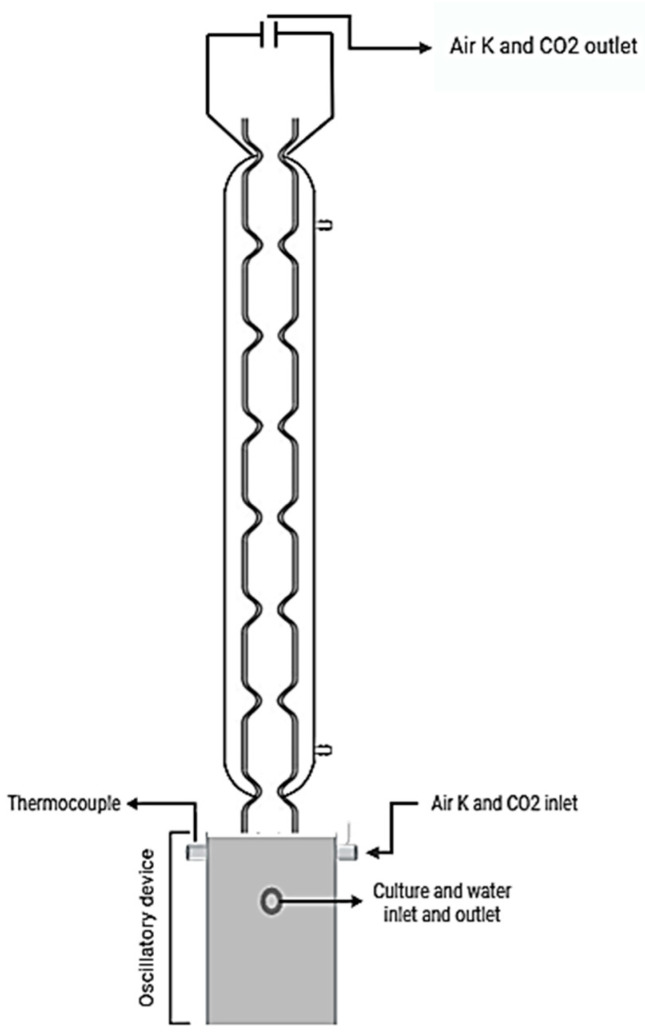
Schematic diagram of the OFR-SPC used in the experiments.

Experiments on microalgae growth were carried out in an OFR-SPC (Figure 1 and Figure A1). This reactor uses controlled oscillatory movement to enhance mixing and mass transfer. By adjusting the frequency and amplitude, it promotes fluid movement from the walls to the centre of a vertical column that contains evenly spaced baffles or holes. This oscillatory flow enhances mass transfer efficiency compared to conventional PBRs and stirred tanks, due to the improved hydrodynamic conditions. When a gas stream, such as CO_2_, is injected into the liquid column, mixing in the liquid phase increases. The concentration gradient between the culture medium and the gas bubbles facilitates CO_2_ transfer from the gas to the liquid, establishing an equilibrium CO_2_ concentration between the gas and liquid phases, according to Equation (1):

(1) $$\frac{dC_{{CO}_{2}L}}{dt}={\left[k_{L}a\right]}_{{CO}_{2}}^{g}(C_{{CO}_{2}L}^{*}-C_{{CO}_{2}L})$$

where ${dC}_{{CO}_{2}L}$*/dt* represents the rate of change of dissolved CO_2_ concentration in the liquid phase (mol m^−3^ h^−1^), ${\left[k_{L}a\right]}_{{CO}_{2}}^{g}$ corresponds to the volumetric mass transfer coefficient for CO_2_ (h^−1^), $C_{{CO}_{2}L}^{*}$ the equilibrium dissolved CO_2_ concentration at the gas–liquid interface (mol m^−3^), $C_{{CO}_{2}L}$ the actual dissolved CO_2_ concentration in the bulk liquid (mol m^−3^), and $\left(C_{{CO}_{2}L}^{*}-C_{{CO}_{2}L}\right)$ represents the concentration gradient.

**Figure A2:**
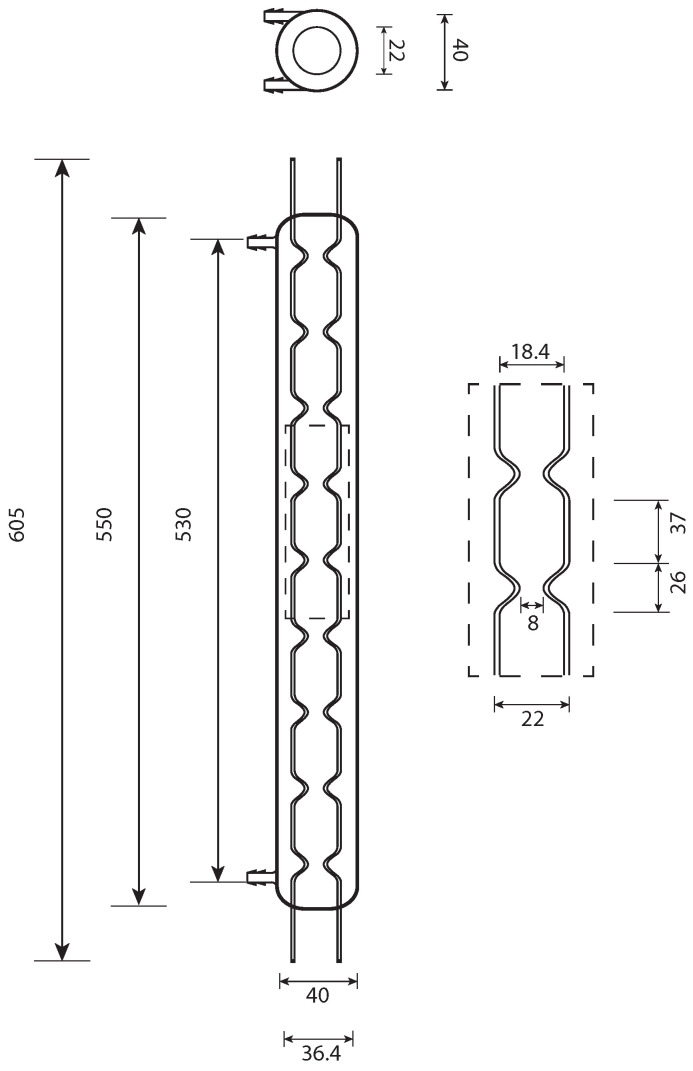
Schematics of the OFR-SPC column—the measurements are given in mm, and the cooling jacket was not utilised in this experiment. (Credit: OFRTECH - Process Intensification, Lda - www.ofrtech.com)

The OFR-SPC offers two key advantages [15]: (i) enhanced gas–liquid mass transfer area: by adjusting the oscillation frequency and amplitude, the OFR can generate smaller bubbles—a more rapid oscillatory motion increases the surface area-to-volume ratio by reducing bubble size, thereby enhancing the available interface for gas exchange; and (ii) prolonged bubble residence time—smaller bubbles experience a higher drag force, relative to larger ones, which counteracts their natural buoyancy, slowing their ascent. The reactor had a total height of 605 mm, an active length of 530 mm, an outer diameter of 40 mm, and an inner diameter of 36.4 mm (Figure A2). The smooth periodic constrictions were spaced 37 mm apart, with a constriction diameter of 8 mm and a maximum tube diameter of 22 mm at the widest point, giving a baffle opening ratio of 0.13. The constriction neck was 26 mm, and the outer wall width was 18.4 mm. The experiments were conducted at a working volume of 120 mL, and the culture was continuously supplied with a total gas flow of 60 cm^3^ min^−1^ (0.5 vvm), consisting of 95% (*v*/*v*) reconstituted air-K (79% of N_2_ and 21% O_2_) and 5% (*v*/*v*) of pure CO_2_. The individual gas streams were regulated using two calibrated mass flow controllers (Alicat Scientific, Tucson, AZ, USA), and the streams were combined prior to entering the reactor through a single bottom gas inlet. Gas exited from the top of the reactor. An LED panel (white light 4000 K) was used to illuminate the culture at an average light intensity of 190 µmol m^−2^ s^−1^. Since the experiment was conducted indoors, the light intensity was measured using a HD2102.2 portable photo-radiometer datalogger (Delta Ohm, Caselle di Selvazzano, Italy) and was considered constant throughout the experiment. A thermocouple connected to the reactor was used to monitor the temperature through the OFRTech software (OFR—UnitControl 1.0 Software). Furthermore, syringes were placed at both the top and bottom of the reactor for sample collection for the subsequent analysis of microalgae growth and sedimentation. The pH of the samples was measured using Consort’s C6010 electrochemical analyser (Brussels, Belgium). Each experiment lasted four days to allow exponential growth of the microalgae. In previous experiments from the research group in the same reactor under similar cultivation conditions, a Design of Experiments (DoE) was conducted to test two factors: frequency and amplitude of oscillation. These tests explored oscillation frequencies of 0.5 and 1.5 Hz and amplitudes of 6 and 12 mm. However, the optimum conditions for microalgae growth and sedimentation were not identified within that range, as the results consistently pointed to the upper boundaries of both variables, suggesting that the optimum lay beyond the tested range (Table S1 and Figure S1). Therefore, based on these results, four conditions were tested in duplicate, combining oscillation frequencies of 0.5 and 2.5 Hz with amplitudes of 6 and 18 mm, representing two independent biological replicates (*n* = 2; A1 and A2). For each biological replicate, the biomass and nutrient concentrations were measured in triplicate at each time point (d). Statistical analysis and parameter calculations were performed using the mean value of technical replicates.

The energy input required to achieve the tested oscillatory conditions was estimated using the quasi-steady flow model (Equation (2)), which accounts for both the mechanical power dissipated by the oscillatory flow and the contribution of rising bubbles (Equation (3)):

(2) $${\left(\frac{P}{V}\right)}_{0}=\frac{2\rho N_{b}}{3\pi C_{D}^{2}}\frac{1-\alpha^{2}}{\alpha^{2}}x_{0}^{3}\omega^{3}$$

(3) $${\left(\frac{P}{V}\right)}_{B}={\rho \mathrm{gu}}_{G}$$

where ρ is the fluid density (kg m^−3^), *N_b_* is the number of baffles per unit length (m^−1^), *C_D_* is the orifice discharge coefficient (taken as 0.7), α is the baffle-free cross-sectional area defined as *(d*_0_*/D)*^2^; $x_{0}$ is the oscillation amplitude (m), ω is the angular frequency of oscillation defined as *2*π*f*, *g* is the gravitational constant (m s^−2^), and $u_{G}$ is the superficial gas velocity (m s^−1^), depending on the frequency and amplitude applied [18]. Table S2 gathers the values used for the power density and energy estimates for the tested oscillatory conditions.

The OFR-SPC has previously demonstrated power density values ranging from 11 to 38 W m^−3^ at low oscillation amplitudes and frequencies, which are considerably lower than those typically reported for conventional tubular PBRs, where power consumption can reach 1100–3300 W m^−3^. Additionally, when compared to bubble-column and flat-plate airlift PBRs, which require power densities of 24–36 W m^−3^ and 16–25 W m^−3^, respectively. These characteristics justify selecting this system as an alternative to conventional sparging and stirring configurations for microalgae cultivation.

### 2.3. Microalgae Growth Monitoring

Approximately 1 mL of sample was removed from the reactor using the syringe in the upper part of the installation, which was connected to a quartz cell joined to a SPEC RES+ UV/Vis spectrometer with an LS-DW Deuterium Tungsten-Halogen light source (Sarspec, Vila Nova de Gaia, Portugal), for subsequent measurement of the optical density (OD) of the culture at 680 nm (OD_680 nm_). The absorbance data from the Sarspec SPEC spectrometers were controlled and analysed using the LightScan spectroscopy software (LightScan 1.0 Software). Distilled water was used as a blank that was read before sample collection. The biomass concentration (dry weight—DW) was calculated from the OD using a previously determined calibration curve (Table S3). To this end, *C. vulgaris* samples were prepared at different concentrations. The OD was measured, and the biomass concentration was determined as described by Mahmoud et al. [19].

Equation (4) can be used to calculate the specific growth rate *µ* (d^−1^) during the exponential growth phase:

(4) $$\mu =\frac{\ln\left(\frac{X_{e,f}}{X_{e,i}}\right)}{t_{e,f}-t_{e,i}}$$

where *X_e,f_* and *X_e,i_* represent the biomass concentration (mg_DW_ L^−1^) at times *t_e,f_* and *t_e,i_,* corresponding to the end and beginning of the exponential growth phase, respectively.

Average biomass productivity (*P_X,avg_*_,_ mg_DW_ L^−1^ h^−1^) was calculated (Equation (5)), where *X_f_* and *X*_0_ represent the biomass concentration at times *t_f_* and *t*_0_, corresponding to the final and initial days of the experiment, respectively. The daily biomass productivity was calculated as described below, using the biomass concentrations and time values from consecutive days.

(5) $$P_{X,\mathrm{avg}}=\frac{X_{f}-X_{0}}{t_{f}-t_{0}}$$

The CO_2_ fixation rate (R_C_) was calculated based on its relationship with the microalgae carbon content (C_C_), biomass productivity and molecular weight of CO_2_ (M_CO2_), and carbon (M_C_) [20]:

(6) $$R_{C}=C_{C}\times P_{X}\times \frac{M_{CO_{2}}}{M_{C}}$$

Considering the typical molecular formula of microalgae biomass, CO_0.48_H_1.83_N_0.11_P_0.01_, each gram of microalgae biomass is equivalent to about 1.88 g of captured CO_2_ [20]. This calculation assumes a fixed biomass elemental composition and a constant carbon content of 51%, which may vary with the cultivation conditions. Therefore, the CO_2_ fixation rates reported here represent theoretical estimates rather than directly measured values.

Values are reported separately for each biological replicate (A1 and A2). *X_max_* is expressed as the mean ± SD of technical triplicates (n = 3). *µ*, *P_X,avg_*, and R_C_ were calculated individually for each assay.

### 2.4. Nutrient Removal

Samples of roughly 4 mL were collected daily and centrifuged in 15 mL Falcon tubes using a CENTROMIX mod. S-549 (J.P. Selecta, Barcelona, Spain) at 4000 rpm for 10 min at 20 °C, and the supernatant was frozen at −20 °C until additional analysis.

The content of nitrate-nitrogen (NO_3_-N) in the samples was ascertained via UV spectroscopy at 220 nm using a T80 UV/VIS spectrometer from PG Instruments (Lutterworth, UK), as described by [14]. The procedure entailed: (i) defrosting the supernatant; (ii) diluting 500 µL of the sample in 9500 µL of distilled water; (iii) filtering the sample using a syringe and 0.22 µm cellulose acetate filters; and (iv) measuring the absorbance in a quartz cuvette at 220 nm (Abs_220nm_). NO_3_-N concentration was calculated through a previously determined calibration curve (Table S3). As the 4 mL daily volume removed for nutrient analysis was not returned to the reactor, the working volume was restored to 120 mL at the start of each day by adding autoclaved distilled water to compensate for both evaporation and nutrient sampling losses.

To determine the concentration of phosphate-phosphorus (PO_4_-P) in the samples, a spectrophotometer (GENESYS 10 UV, Thermo Scientific, Waltham, MA, USA) was used to measure the absorbance at 820 nm of the phosphomolybdate complex, which is created when ammonium molybdate reacts with inorganic phosphate in the presence of ascorbic acid [21]. For this purpose, 700 µL of a mixture containing ammonium molybdate and ascorbic acid was combined with 300 µL of the supernatant. The samples were then incubated for one hour at 37 °C in the dark, and the absorbance was measured (Abs_820nm_). The PO_4_-P concentration was calculated through a previously determined calibration curve (Table S3).

Mass removal (MR), in mg L^−1^; removal rate (RR), in mg L^−1^ d^−1^; removal efficiency (RE%); and specific biomass yield (Y_X/S_, mg_X_ mg_S_^−1^) were calculated according to Equations (7)–(10), respectively:

(7) $$\mathrm{MR}=S_{0}-S_{f}$$

(8) $$\mathrm{RR}=\frac{\mathrm{MR}}{t_{f}-t_{0}}$$

(9) $$\mathrm{RE}=\frac{\mathrm{MR}}{S_{0}}\times 100$$

(10) $$Y_{X/S}=\frac{X_{f}-X_{0}}{S_{0}-S_{f}}$$

where *S*_0_ corresponds to the nutrient concentration at the beginning of the experiment, *t*_0_, and *S_f_* to the final nutrient concentration at the end of the experiment, *t_f_*.

Each oscillatory condition was performed in duplicate (A1 and A2), representing independent biological replicates. At each day of the experiment, nutrient concentrations were measured in triplicate, and results are expressed as the mean ± standard deviation of technical replicates (n = 3). Mass removal (MR), removal efficiency (RE), and removal rate (RR) were calculated using the mean nutrient concentrations obtained at the initial (*t*_0_) and final (*t_f_*) time points of each assay.

### 2.5. Sedimentation Kinetics and Efficiency

Sedimentation kinetics were assessed daily using the same 1 mL sample collected from the upper part of the reactor, as described in Section 2.3, which was retained in the quartz cuvette connected inline to the SPEC RES+ UV/Vis spectrometer for a 2-h period. During this time, OD_680_ was continuously recorded as cells settled within the cuvette. After the 2-h measurement, the sample was returned to the reactor. A relative OD ratio was calculated by normalising each measurement to the initial value (OD_0_) for that period, ensuring all curves started from a common reference point. Sedimentation efficiency (SE) was determined using Equation (11), where *OD_s,_*_0_ represents the initial optical density and *OD_s,f_* is the final measurement of the 2-h interval [22].

(11) $$\mathrm{SE}\left(\%\right)=\frac{{\mathrm{OD}}_{s,0}-{\mathrm{OD}}_{s,f}}{{\mathrm{OD}}_{s,0}}\times 100$$

The experimental data on the OD ratio at 680 nm over time were fitted to the modified Gompertz model (Equation (12)), where *k* is the sedimentation kinetic constant (h^−1^) and *λ* is the lag time (h). These parameters were calculated by minimising the sum of squared residuals using the Solver supplement of Microsoft Excel. The root mean squared error (RMSE) and coefficient of determination (R^2^) were calculated using Equations (13) and (14), respectively, to evaluate the performance of the calculated models:

(12) $${\mathrm{OD}}_{s,f}={\mathrm{OD}}_{s,0}+\left({\mathrm{OD}}_{s,f}-{\mathrm{OD}}_{s,0}\right)\times \exp\left(-\exp\left(k\left(\lambda -t\right)+1\right)\right)$$

(13) $$\mathrm{RMSE}=\sqrt{\frac{\sum_{i=1}^{n}{\left(y_{i}-{\hat{y}}_{i}\right)}^{2}}{n}}$$

(14) $$R^{2}=1-\frac{\sum_{i=1}^{n}{\left(y_{i}-{\hat{y}}_{i}\right)}^{2}}{\sum_{i=1}^{n}{\left(y_{i}-\overset{-}{y}\right)}^{2}}$$

where the variable *y* corresponds to the experimental values, $\hat{y}$ is the predicted model values, $\bar{y}$ is the average experimental values and *n* is the data size. The quality of the model fit improves when the parameters RMSE and R^2^ approach 0 and 1, respectively.

The modified Gompertz model was selected because the experimental sedimentation profiles exhibited a clear lag phase prior to the onset of a decrease in biomass concentration. Classical first- and second-order kinetic models assume immediate decay at *t* = 0 and therefore cannot represent this induction period. The Gompertz model allows for independent quantification of the lag time (*λ*), corresponding to the aggregation/structural rearrangement phase preceding effective settling, and the maximum sedimentation rate (*k*), associated with the highest biomass removal velocity. This sigmoidal formulation provides superior fitting performance and better physical interpretability of the sedimentation dynamics [23,24].

In addition, the zeta potential was also analysed daily, a parameter that made it possible to identify the colloidal dispersion’s stability, since smaller absolute values of zeta potential may indicate a higher tendency for cell aggregation, and consequently a higher tendency to settle. These experiments were performed directly in the culture medium, without prior washing or resuspension in a low-ionic-strength buffer, to reflect the actual electrostatic surface conditions experienced by the cells during sedimentation in the reactor. For that purpose, 1 mL of culture was collected from the reactor, placed in a DTS1070 folded capillary cell used in a ZetaSizer Nano ZS (Malvern Instruments, Worcesterhire, UK), and the zeta potential of the sample was read using ZetaSizer software (version 8, Malvern).

### 2.6. Statistical Methods and Models

In this study, five explanatory variables were considered with the experimental data collected from day 1 to day 4: (i) cultivation time (*x*_1_, h); (ii) oscillation amplitude (*x*_2_, mm); (iii) oscillation frequency (*x*_3_, Hz); (iv) sedimentation kinetic constant on day 0 (*x*_4_, h^−1^), which refers to the condition of the microalgae culture at the time it is introduced into a new culture medium or reactor, defined by the sedimentation kinetic constant (*k*) at day 0; and (v) zeta potential (*x*_5_, mV). Multicollinearity among explanatory variables was assessed using a Pearson correlation matrix (significance level *α* = 0.05, *r_crit_* = ±0.329, degrees of freedom = 34).

Multiple linear regression (MLR) models the relationship between explanatory variables and response variables by fitting a linear equation to the observed data. The dependent variable (*y*) and the predicted value given by the regression model ($\hat{y}$) were determined by the following equations, where *x_i_* (i = 1, …, k) are the explanatory independent variables, $\hat{\beta_{i}}$ (i = 0, …, k) are the regression coefficients, and ϵ is the error associated with the regression.

(15) $$y=\hat{\beta_{0}}+\sum_{i=1}^{k}\hat{\beta_{1}}x_{i}+\epsilon$$

(16) $$\hat{y}=\hat{\beta_{0}}+\sum_{i=1}^{k}\hat{\beta_{1}}x_{i}$$

Five dependent variables were analysed: (i) sedimentation kinetics (*y*_1_, h^−1^) defined by the sedimentation constant (*k*) from days 1 to 4; (ii) sedimentation efficiency (*y*_2_, %); (iii) daily biomass productivity (*y*_3_, mg L^−1^ d^−1^); (iv) nitrogen removal rate (*y*_4_, mg L^−1^ d^−1^); and (v) phosphorous removal rate (*y*_5_, mg L^−1^ d^−1^).

The principal component analysis (PCA) was applied to remove the dependence on the explanatory variables through the creation of new variables, named principal components (PCs), that are orthogonal and uncorrelated. Furthermore, a principal component regression (PCR) was performed, combining the linear Equations (15) and (16) with the PCA, using PCs as input variables.

The dataset used for MLR and PCR comprised a limited number of experimental observations (n = 2 biological replicates across 4 time points per condition), which constrains the statistical power of the models and increases the risk of overfitting. The model outputs should therefore be interpreted as exploratory rather than confirmatory. Model assumptions of the normality of residuals were not formally tested due to the limited sample size, which limits the present statistical analysis and should be considered in future work.

## 3. Results and Discussion

### 3.1. Growth Monitoring

The overall average temperature and pH were 29 ± 2 °C and 6.9 ± 0.4, respectively. Initially, the pH values were similar across all experiments, ranging from 7 to 8. On days 0 and 1, the pH levels remained stable; thereafter, they decreased. A decrease in pH over time is expected, as when dissolved, CO_2_ reacts with water to form H_2_CO_3_, which dissociates into H^+^ and HCO_3_^−^. Throughout all of the tests, the values remained in the 6–8 range, which is in line with the results obtained by Chowdury et al. [25]. The pH dynamics observed throughout the cultivation period reflect the balance between two opposing processes: the dissolution of CO_2_, which generates carbonic acid and drives the medium acidification, and the assimilation of nitrate by *C. vulgaris*, which consumes protons and promotes alkalinisation of the culture medium [26]. In batch mode under different light intensities and temperatures, the authors concluded that *Scenedesmus* sp. LX1 tended to grow at intermediate pH values (7–9), whereas extreme pH values (5 and 11) inhibited growth.

**Figure 2:**
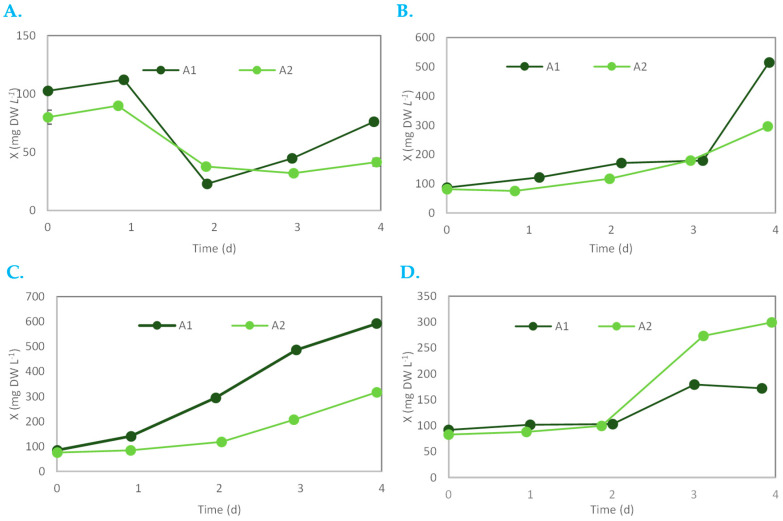
Time-course evolution of biomass concentration under different oscillatory conditions: (**A**) 0.5 Hz|6 mm; (**B**) 0.5 Hz|18 mm; (**C**) 2.5 Hz|6 mm; (**D**) 2.5 Hz|18 mm. Each condition was performed in duplicate (A1 and A2), representing independent biological replicas.

The influence of oscillation frequency and amplitude on *C. vulgaris* growth and CO_2_ fixation in the oscillatory flow reactor (OFR) was systematically evaluated (Figure 2 and Table 1). The results revealed that both parameters significantly impacted the microalgae performance, confirming the hydrodynamic sensitivity of microalgae cultures. Under low oscillatory conditions (0.5 Hz|6 mm), the average biomass productivity was negative in both assays, indicating biomass loss or underestimation due to sedimentation at the reactor base. This low-shear environment likely resulted in insufficient mixing and mass transfer, limiting CO_2_ bioavailability and nutrient distribution. At low oscillation intensity, the energy transmitted is insufficient to generate fully developed vortices between constrictions, resulting in poorly mixed zones where cells settle, and CO_2_ gradients persist. This is consistent with the observed biomass sedimentation and near-zero nutrient removal under these conditions [14,15]. No correction was applied to the biomass measurements under these conditions. For that reason, the biomass concentration and productivity values reported for 0.5 Hz|6 mm are likely to underestimate the true biomass due to cell accumulation at the reactor base rather than reflecting actual growth inhibition.

Conversely, increasing the amplitude to 18 mm at the same frequency (0.5 Hz) significantly enhanced the growth parameters. The highest specific growth rate (μ = 1.31 d^−1^) was recorded under these conditions, along with a marked increase in maximum biomass concentration (514.9 mg_DW_ L^−1^), average biomass productivity (4.55 mg L^−1^ h^−1^), and CO_2_ fixation rate (8.34 mg L^−1^ h^−1^). This suggests that increased amplitude enhanced fluid displacement through the constrictions sufficiently to overcome dead zones and improve CO_2_, dissolution, and nutrient distribution, without introducing excessive shear stress on the cells [15]. However, a large discrepancy was observed between replicates at this condition (µ = 1.31 vs. 0.446 d^−1^), which may reflect differences in the inoculum physiological state, residual biofilm accumulation at the reactor base between runs, or partial biomass settling at low frequency, reducing the representativeness of OD measurements collected from the upper part of the reactor. At 2.5 Hz|6 mm, the system achieved the highest biomass concentration (X_max_ = 592.0 mg_DW_ L^−1^), average biomass productivity (5.36 mg L^−1^ h^−1^), and CO_2_ fixation rate (R_C_ = 9.82 mg L^−1^ h^−1^), indicating that increased oscillation frequency significantly enhances microalgae growth. Mechanistically, higher frequencies lead to more frequent vortex formation at the constrictions, reducing the bubble size and increasing the gas–liquid interfacial area, thereby improving the CO_2_ mass transfer and availability to cells [15]. Furthermore, more frequent oscillations promote shorter light/dark cycles, as cells circulate through illuminated and shaded zones, which has been shown to enhance photosynthetic efficiency in microalgae [27]. Reduced sedimentation at higher frequencies also ensures that cells remain in suspension, maintaining access to both light and nutrients. These findings are consistent with those of Vale [28], who observed an increase in kLa in the OFR-SPC with increasing oscillation amplitude and frequency. The author observed the highest average *C. vulgaris* productivities (6.02 mg_DW_ L^−1^ h^−1^, corresponding to μ = 0.369 d^−1^) in the assays with 1% (*v*/*v*) CO_2_ at 1.5 Hz|19 mm. Despite slightly higher average biomass productivities, the specific growth rates were lower than those achieved in the current study.

In contrast, when both amplitude and frequency were simultaneously increased (2.5 Hz|18 mm), the microalgae growth parameters exhibited contrary behaviours: while specific growth rates increased (0.552 and 0.811 d^−1^), the average biomass productivity decreased (0.870 and 2.28 mg_DW_ L^−1^ h^−1^). The decrease in biomass productivity may be attributed to excessive oscillation intensity, which can disrupt photosynthetic activity and cell division or increase metabolic energy expenditure on recovery rather than growth. This would explain why growth rates were maintained while net biomass accumulation declined. Direct confirmation of cell viability would require membrane integrity assays, such as Evans blue dye staining [29]. Additionally, photosynthetic performance indicators such as chlorophyll fluorescence (Fv/Fm) [30] could provide complementary information on the physiological state of the cells. Both are recommended for future work. Overall, these findings confirm that high frequencies (2.5 Hz) combined with lower amplitude (6 mm) provide an optimal balance between mixing efficiency and cell stability. These hydrodynamic conditions likely promote the formation of small gas bubbles with higher residence times, as previously described by [15], thereby enhancing CO_2_ absorption and supporting robust growth [31,32,33]. The maximum specific growth rate (μ = 1.31 d^−1^) and biomass productivity (~5.36 mg_DW_ L^−1^ h^−1^) were within the range reported in the literature for *C. vulgaris* cultivated under comparable conditions, although higher growth rates have been reported for other *Chlorella* strains and systems [9,17]. Nad’ et al. [9] reported biomass productivities of 0.51 g L^−1^ d^−1^ at the laboratory scale, declining to 0.13 g L^−1^ d^−1^ at the pilot scale, with CO_2_ fixation rates of 0.95 and 0.25 g L^−1^ d^−1^, respectively, reinforcing the efficacy of OFR-SPC as a promising bioreactor design for enhanced CO_2_ capture and biomass production. The outlet gas CO_2_ concentration was not measured in this study. However, CO_2_ consumption was indirectly assessed through biomass productivity, as carbon fixation is directly coupled to biomass accumulation via photosynthesis. Direct quantification of CO_2_ fixation through gas-phase analysis is recommended for future work.

### 3.2. Nutrient Removal

**Figure 3:**
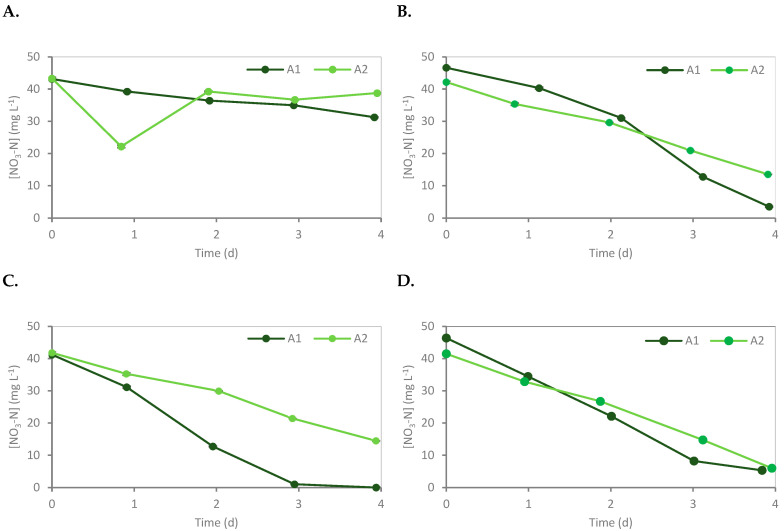
Time-course evolution of NO_3_-N concentration under different oscillatory conditions: (**A**) 0.5 Hz|6 mm; (**B**) 0.5 Hz|18 mm; (**C**) 2.5 Hz|6 mm; (**D**) 2.5 Hz|18 mm. Each condition was performed in duplicate (A1 and A2), representing independent biological replicas.

**Figure 4:**
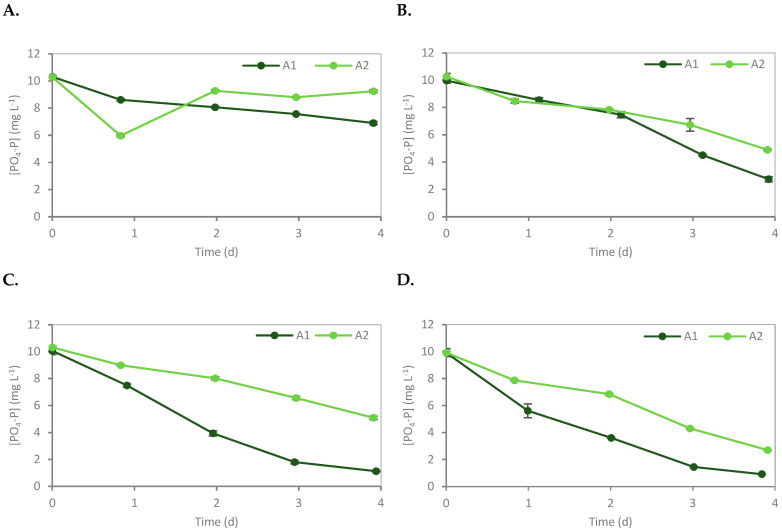
Time-course evolution of PO4-P concentration under different oscillatory conditions: (**A**) 0.5 Hz|6 mm; (**B**) 0.5 Hz|18 mm; (**C**) 2.5 Hz|6 mm; (**D**) 2.5 Hz|18 mm. Each condition was performed in duplicate (A1 and A2), representing independent biological replicas.

The results showed that nitrogen and phosphorus concentrations decreased almost linearly over time in most experiments (Figure 3 and Figure 4). However, this trend was not observed in the assays operated at 0.5 Hz|6 mm, where the nutrient concentrations remained nearly constant after the first day of the experiment. These patterns are consistent with the biomass growth results, as these assays exhibited a decline in biomass concentration, indicating that nutrients were not being assimilated by the microalgae. At 0.5 Hz|18 mm, a non-linear nutrient removal trend was observed, with removal rates declining after the initial days, despite relatively high biomass concentrations. This may be linked to partial biomass settling under low-frequency conditions, where settled cells at the reactor base have reduced access to nutrients in the bulk liquid, limiting the overall assimilation efficiency even when the total biomass appears adequate. Higher oscillation frequency improves nutrient removal through two complementary mechanisms. First, more frequent vortex renewal at the constrictions reduces the thickness of the boundary layer surrounding each cell, increasing the diffusion gradient for NO_3_-N and PO_4_-P from bulk liquid to the cell surface. Second, enhanced mixing promotes higher metabolic activity by improving CO_2_ availability and light exposure, thereby increasing cellular demand for nitrogen and phosphorus as building blocks for biomass synthesis [14]. Even when bulk concentrations were still detectable, inadequate mixing at 0.5 Hz|6 mm might have produced nutrient-depleted microenvironments around settled cell aggregates, further reducing intake [14,15].

The initial NO_3_-N and PO_4_-P concentrations in the OECD medium were approximately 41.2 mg L^−1^ and 10.3 mg L^−1^, respectively, corresponding to an N:P molar ratio of 9:1 and served as the reference values for the removal efficiencies reported below. Table 2 shows the nutrient removal parameters for each assay, highlighting the effect of oscillatory variables on nitrogen and phosphorus removal. For nitrogen, the mass removal, removal rate, and removal efficiency ranged from 4.57 to 43.2 mg L^−1^, 1.16 to 11 mg L^−1^ d^−1^, and 10.5 to 100%, respectively. The lowest nitrogen removal parameters were verified at 0.5 Hz|6 mm. In contrast, at 0.5 Hz|18 mm, the highest mass removal and removal rate were achieved, corresponding to the highest specific growth rate and maximum biomass concentration. Removal efficiencies were generally high (with half of the assays above 80%); however, this parameter is strongly influenced by the feed N:P feed ratio [34] and the nutrient requirements of the microalgae under specific cultivation conditions, which ultimately affect biomass composition. For phosphorus, the mass removal, removal rate, and removal efficiency ranged from 1.04 to 8.99 mg L^−1^, 0.27 to 2.34 mg L^−1^ d^−1^, and 10.1% to 90.7%, respectively. The highest values for all three parameters were achieved at 2.5 Hz|18 mm, while the lowest occurred at 0.5 Hz|6 mm. The oscillatory conditions yielding the maximum nitrate and phosphorus removal differed in amplitude, likely reflecting the distinct metabolic pathways governing their uptake, with nitrate assimilation being more tightly coupled to photosynthetic activity, while phosphorus removal may be more sensitive to mixing intensity and consumption dynamics.

Regarding specific biomass yields, negative values were observed at 0.5 Hz|6 mm due to a decrease in biomass concentration during the assays. Nitrogen and phosphorus are two crucial macronutrients in microalgae biomass [35]: (i) nitrogen content ranges from 1 to 14% of dry weight, with typical values between 5 and 10%; and (ii) phosphorus content varies from 0.05% to 3.3%. Since the specific biomass yield corresponds to the inverse of the nutrient mass fraction in biomass, typical values range from 7 to 100 mg_X_ mg_S_^−1^ for nitrogen and 30 to 2000 mg_X_ mg_S_^−1^ for phosphorus. For experiments at 2.5 Hz|18 mm, lower values were achieved for both elements, suggesting that less biomass was produced with the same mass of nutrient removed from the medium. This outcome may be explained by the increased sedimentation of biomass in the OFR-SPC under stronger oscillatory conditions, which could have affected the measurement of biomass produced in these assays.

### 3.3. Sedimentation Kinetics and Efficiency

Table 3 presents the daily zeta potential, microalgae sedimentation efficiency after 2 h of settling, and the kinetic parameters determined through modified Gompertz model fitting. The initial zeta potential values were similar between the experiments, around −30 mV. Gonçalves et al. [24] reported zeta potential values of microalgae and cyanobacteria ranging from (−35.4  ±  0.4) to (−48.1  ±  0.9) mV. At high absolute zeta potential values, electrostatic repulsive forces exceed the van der Waals attractive forces, thereby maintaining cells in a dispersed state. However, their ability to form cell aggregates is strongly influenced by the ionic strength of the culture medium.

Overall, the Gompertz model provided a good fit to the experimental data (fitting curves shown in Figure S2), with RMSE values close to 0. However, even though most R^2^ values were close to 1, moderate R^2^ values indicate that the model may not fully explain the data’s variability due to high noise in the OD measurements from the cell suspensions. After 2 h of settling, the maximum lag time observed was (0.80 ± 0.01) d on day 0 in the 0.5 Hz|6 mm assay. In contrast, most assays exhibited low lag times, often zero, indicating that sedimentation began early in the settling process. After one day of cultivation, the absolute zeta potential remained high and far from zero, preventing cell aggregation and accounting for the lower k values observed during this period (slower sedimentation). These results suggest that the culture was in a lag phase, providing stability rather than exponential growth [36].

After 2–3 days, an increase in the sedimentation rate was observed in all experiments. In some conditions, notably at 0.5 Hz|6 mm, this coincided with a reduction in the absolute zeta potential over time, suggesting weakened electrostatic repulsion and increased tendency for aggregation. However, this trend was not consistent across all experimental conditions, suggesting that mechanisms beyond surface charge may also contribute to the observed sedimentation behaviour. In cases where the zeta potential decreased, the electrostatic repulsion between particles became weaker, and the microalgae began to aggregate and flocculate, forming larger particles that, according to Stokes’ Law, have higher settling velocities. This aggregation behaviour could potentially be further enhanced by EPS secretion, which has been reported to increase progressively with cultivation time and in response to mechanical stimulation. EPSs are surface-active molecules that adsorb onto cell surfaces, reducing the absolute zeta potential and screening electrostatic repulsion, thereby promoting cell–cell contact and bioflocculation [37]. Under oscillatory conditions, repeated mechanical stimulation of cells passing through the constrictions may accelerate EPS release, progressively modifying the cell surface charge and increasing the aggregate size over the cultivation period. However, EPSs were not quantified in the present study, and this mechanism remains hypothetical. This is consistent with the PCR results, which showed a positive influence of cultivation time on the sedimentation kinetic constant, likely reflecting cumulative EPS accumulation over the 4-day experiment. The oscillatory conditions also determine the magnitude of shear stress experienced by cells, which directly influences aggregate stability and sedimentation behaviour. At low frequencies (0.5 Hz), shear forces are minimal, allowing aggregates to form and persist, consistent with the high sedimentation efficiencies observed at 0.5 Hz|6 mm. Conversely, at 2.5 Hz|18 mm, the combination of high frequency and large amplitude likely generates substantially higher shear forces, disrupting forming aggregates and reducing sedimentation efficiency despite promoting growth. This is consistent with the findings by Li et al. [38], who reported that hydrodynamic turbulence enhanced the autoflocculation efficiency of *C. vulgaris* by 40–53.3% under moderate shear, achieving maximum floc sizes of 373.5 ± 36.4 µm and settling velocities of 2.17 ± 0.29 m/h. However, once shear strength exceeded 0.0115 N/m^2^, floc disaggregation was observed, demonstrating that excessive turbulence is detrimental to aggregate formation. Although shear stress was not directly measured in this study, qualitative estimates based on the oscillatory Reynolds number suggest that higher-frequency, higher-amplitude combinations generate more turbulent flow regimes, as reported for similar OFR-SPC geometries [14,15]. It should also be noted that the observed decline in sedimentation efficiency under high oscillatory conditions could reflect either mechanical cell damage, reducing the aggregate-formation capacity, or a genuine shift in flocculation behaviour driven by surface charge changes. Finally, by day 4, the sedimentation slowed again in the assays with the most extreme conditions: 0.5 Hz|6 mm and 2.5 Hz|18 mm. In the later experiment, the zeta potential increased from day 3 to 4, which may be related to a change in the medium’s ionic strength. Gonçalves et al. [24] concluded that ionic strength should be considered when studying microalgae sedimentation, as it influences surface charge and can increase or decrease electrostatic repulsions. As microalgae grow, they consume the nutrients supplied to the culture in ionic form, reducing the dissolved ion concentration and gradually lowering the medium’s ionic strength. Thielemans et al. [39] demonstrated that ionic strength directly affects floc size and separation efficiency in *C. vulgaris*, with freshwater cultures forming flocs of approximately 390 µm and achieving separation efficiencies as high as 99% via gravity sedimentation. Additionally, the culture age must be considered, as ageing cultures are characterised by changes in cell physiology, including reduced metabolic activity, increased cell death, and alterations in cell surface properties, all of which may affect cells’ tendency to aggregate and sediment. Together, these factors may explain why, on the last day of cultivation, when nutrient concentrations were lower and the culture had reached a more advanced physiological stage, the sedimentation tendency decreased.

Sedimentation efficiencies were lowest in the 0.5 Hz|18 mm and 2.5 Hz|6 mm assays, ranging from 16.8–43.0% and 22.8–50.2%, respectively. Variations in the values could also be explained by factors such as temperature fluctuations, differences in initial growth phases, or variations in ion concentrations in the culture medium. Moreover, the presence of organic contaminants on the microalgae surface or residual chemicals can also contribute to changes in the microalgae’s surface charge [40]. Rao et al. [41] reported a maximum gravity efficiency of 87% for *C. vulgaris* under optimised bench-scale flocculation conditions while noting that sedimentation without chemical assistance tends to produce a low sludge solid content (~1.1%) and proceeds slowly. Conversely, conditions 0.5 Hz|6 mm provided the highest SE, 92.3% and 100%, during the second and third days, respectively. Kim and Kwak [42] concluded that auto/bioflocculation conditions induced by pH and mixing stresses can improve separation efficiency in gravity sedimentation and achieved removal efficiencies of over 90% for *C. vulgaris* using dissolved air flotation and dissolved CO_2_ flotation. In the same previous study, the highest sedimentation efficiency (48.5%) was promoted under low amplitude (6 mm) and high frequency (1.5 Hz). This emphasises that increasing the oscillation frequency significantly enhances the sedimentation efficiency, demonstrating that these conditions are favourable for efficient sedimentation. In the overall scenario, microalgae cultivation at 2.5 Hz|18 mm showed results that, even at the highest, are favourable for both sedimentation and growth, including nutrient uptake.

### 3.4. Multiple Linear Regression Model

Table 4 shows the statistically significant MLR parameters for each dependent variable (sedimentation kinetic constant, sedimentation efficiency, daily biomass productivity, nitrogen removal rate, phosphorous removal rate) in terms of each explanatory variable (cultivation time, oscillation amplitude, oscillation frequency, sedimentation kinetic constant on day 0—k_to_, zeta potential), using a significance level of 0.05.

For all of the dependent variables, the oscillation amplitude (*β*_2_) was not statistically significant for any of the dependent variables. For the sedimentation kinetics, no explanatory variable was significant; only the constant term (β_0_) remained valid. The zeta potential (*β*_5_) had a positive effect on sedimentation efficiency (*y*_2_), as expected, because higher values promote greater aggregation and flocculation, thereby facilitating the sedimentation of biomass. For the same reason, it was also expected that a negative *β*_5_ regression parameter for average biomass productivity (*y*_3_) would be observed. Considering the same dependent variables, all other regression parameters were positive, with the strongest effect observed for oscillation frequency (β_3_ = 0.60 ± 0.39).

Regarding the nutrient removal rates, oscillation frequency (β_3_) and zeta potential (β_5_) were identified as the main influencing factors, exerting positive and negative effects, respectively. Additionally, MLR revealed a negative correlation between phosphorus removal rate and cultivation time. This negative correlation between zeta potential and nutrient removal rates helps explain the discrepancies observed between experimental runs in the 2.5 Hz and 6 mm condition (Figure 2C and Figure 3C). Despite similar growth rates across replicates, run A1 showed higher X_max_ and P_max_ values, along with lower sedimentation efficiencies, suggesting that cells remained more dispersed in suspension. The same logic applies to Figure 3C,D.

It should be noted that the Pearson correlation matrix indicated no strong pairwise correlations among predictors at α = 0.05. However, since formal V|F values were not calculated, residual multicollinearity cannot be fully excluded. Consequently, these results should be interpreted as exploratory.

### 3.5. Principal Component Regression Model

Table 5 shows the results of the varimax rotation on the three PCs and the corresponding cumulative variance percentages. Three PCs were selected based on the Kaiser criterion (eigenvalues above 1), with eigenvalues of 1.63, 1.22, and 1.00, accounting for about 77% of the original data variance. Bolded values represent the explanatory variables’ primary contributions to each PC. The first principal component (PC1), explaining 32.5% of the variance, is related to oscillation amplitude and sedimentation kinetic constant on day 0 (kt_0_) variables. PC2, which explained 24.4% of the variance, strongly depends on oscillation frequency and zeta potential. Finally, PC3, explaining 20.0% of the variance, had only cultivation time as a variable with an important contribution.

Table 6 presents the statistically significant regression parameters for each dependent variable with respect to each explanatory variable, at the 0.05 significance level, using a PCR model. This model produced some differences compared with the MLR. The sedimentation kinetic constant increased with cultivation time, likely due to the accumulation of EPSs in the culture, which enhances microalgae cell aggregation. In terms of efficiency, oscillation frequency had a negative effect, while zeta potential had a positive effect—consistent with the MLR results. Average biomass productivity decreased with higher oscillation amplitude and zeta potential, but increased with a favourable sedimentation kinetic constant on day 0 and higher oscillation frequency. Nutrient (N and P) removal rates followed a similar trend: they increased with increasing oscillation frequency but decreased with higher zeta potential and longer cultivation times. After applying the PCR model, which accounts for collinearity among explanatory variables, a clearer, more reliable relationship emerged between the experimental conditions (amplitude and frequency). As with the MLR model, the small sample size (n = 2 biological replicates, 4 time points per condition) means that the PCR outputs carry a significant risk of overfitting and should be interpreted as exploratory.

## 4. Conclusions

This study demonstrated the potential of OFR-SPC to intensify microalgae cultivation and biomass harvesting. Results confirmed that oscillation frequency and amplitude strongly influence nutrient removal and sedimentation dynamics, and are likely to influence CO_2_ mass transfer, as previously characterised by Gonçalves et al. [14]. Optimal growth and productivity were obtained at intermediate-to-high frequencies (2.5 Hz) and moderate amplitudes (6 mm), while low-frequency conditions (0.5 Hz|18 mm) also promoted high growth rates through enhanced bulk mixing. Nutrient removal efficiencies for nitrogen (up to 100%) and phosphorus (up to 91%) highlighted the system’s potential for wastewater treatment integration. Sedimentation kinetics were well-fitted to the Gompertz model, with sedimentation efficiencies reaching 100% under favourable conditions, demonstrating the feasibility of simplified biomass recovery. Statistical modelling (MLR and PCR) confirmed the dominant roles of oscillation conditions and zeta potential. However, biomass measurements at 0.5 Hz|6 mm are likely underestimated due to cell settling at the reactor base, and the large replicate variability observed at 0.5 Hz|18 mm limits statistical confidence at this condition. CO_2_ fixation rates represent theoretical estimates based on a fixed biomass elemental composition rather than direct measurement. Finally, shear stress was not directly quantified, and cell health indicators such as chlorophyll fluorescence were not measured, preventing a definitive distinction between mechanical cell damage and flocculation behaviour as drivers of sedimentation trends. Future work should address these limitations by quantifying the oscillatory Reynolds number and shear stress across broader operating ranges, measuring cell health indicators (Fv/Fm, membrane integrity) and EPS secretion to confirm the proposed mechanisms, and investigating the effects of nitrogen and phosphorus ratios on growth and removal efficiency. Improving CO_2_ utilisation through coupled cultivation systems that better align mass transfer kinetics with microalgae metabolic rates represents another key avenue, alongside exploring bioflocculation strategies such as chitosan addition or bacteria co-cultures to enhance sedimentation and biomass recovery, as essential steps towards industrial implementation. Further studies should also validate the model-predicted optimum for the scale-up of the OFR-SPC to pilot scale and include a techno-economic assessment to evaluate the commercial viability of this system for simultaneous microalgae cultivation, nutrient removal, and passive biomass harvesting. Finally, a comparative study with conventional non-oscillatory reactors to quantify relative performance gains should also be addressed in future projects.

## Figures and Tables

**Table 1:** Growth parameters determined for *Chlorella vulgaris* in each experiment.

Experimental Conditions	*µ* (d^−1^)	*X_max_* (mg_DW_ L^−1^)	*P_X,avg_* (mg_DW_ L^−1^ h^−1^)	*R_C_* (mg CO_2_ L^−1^ h^−1^)
0.5 Hz|6 mm|A1	ND	112.1 ± 0.9	<0	<0
0.5 Hz|6 mm|A2	ND	89.91 ± 0.18	<0	<0
0.5 Hz|18 mm|A1	1.31	514.9 ± 4.5	4.55	8.34
0.5 Hz|18 mm|A2	0.446	296.3 ± 0.4	2.29	4.20
2.5 Hz|6 mm|A1	0.493	592.0 ± 2.9	5.36	9.82
2.5 Hz|6 mm|A2	0.435	316.8 ± 3.4	2.55	4.67
2.5 Hz|18 mm|A1	0.552	179.2 ± 0.9	0.870	1.59
2.5 Hz|18 mm|A2	0.811	299.1 ± 2.6	2.28	4.18

ND—not determined due to biomass sedimentation; *P_X,avg_*—average biomass productivity; *R_C_*—CO_2_ fixation rate; *X_max_*—maximum biomass concentration (values are presented as the mean ± standard deviation obtained from the first 3 values of each experiment); *µ*—specific growth rate.

**Table 2:** Nutrient removal parameters for nitrogen and phosphorus.

Nutrients	Experimental Conditions	*MR* (mg L^−1^)	*RR* (mg L^−1^ d^−1^)	*RE* (%)	*Y_X/S_* (mg_X_ mg_S_^−1^)
NO_3_-N	0.5 Hz|6 mm|A1	11.9	3.03	27.5	<0
	0.5 Hz|6 mm|A2	4.57	1.16	10.5	<0
	0.5 Hz|18 mm|A1	43.2	11.0	92.5	9.92
	0.5 Hz|18 mm|A2	28.6	7.32	67.9	7.50
	2.5 Hz|6 mm|A1	41.2	10.5	100	12.3
	2.5 Hz|6 mm|A2	27.3	6.94	65.3	8.82
	2.5 Hz|18 mm|A1	41.1	10.7	88.5	1.95
	2.5 Hz|18 mm|A2	35.5	8.98	85.6	6.09
PO_4_-P	0.5 Hz|6 mm|A1	3.41	0.87	33.0	<0
	0.5 Hz|6 mm|A2	1.04	0.27	10.1	<0
	0.5 Hz|18 mm|A1	7.22	1.84	72.4	59.3
	0.5 Hz|18 mm|A2	5.36	1.37	52.3	40.0
	2.5 Hz|6 mm|A1	8.93	2.27	88.7	56.8
	2.5 Hz|6 mm|A2	5.22	1.33	50.6	46.2
	2.5 Hz|18 mm|A1	8.99	2.34	90.7	8.91
	2.5 Hz|18 mm|A2	7.22	1.85	72.7	30.0

*MR*: mass removal; NO_3_-N: nitrate-nitrogen; PO_4_-P: phosphate-phosphorus; *RE*: removal efficiency; *RR*: removal rate; *Y_X/S_*: biomass yield.

**Table 3:** Sedimentation efficiency (SE), zeta potential, and kinetic parameters estimated using the modified Gompertz model for each experimental condition.

Experimental Conditions	t_c_ (h)	*SE* (%)	*k* (h^−1^)	*λ* (h)	R^2^	RMSE	ζ (mV)
0.5 Hz|6 mm|A1	0	25.2	0.55 ± 0.18	0.28 ± 0.09	0.741	0.065	−30.1 ± 1.25
	21.9	52.2	0.95 ± 0.07	0.00 ± 0.06	0.933	0.044	−28.3 ± 2.15
	46.0	92.3	1.76 ± 0.07	0.00 ± 0.04	0.916	0.087	−21.9 ± 0.73
	70.5	71.3	1.92 ± 0.06	0.00 ± 0.03	0.930	0.059	−19.3 ± 0.87
	94.1	68.1	1.59 ± 0.05	0.00 ± 0.03	0.947	0.047	−18.6 ± 1.62
0.5 Hz|6 mm|A2	0	67.6	2.34 ± 0.05	0.80 ± 0.01	0.930	0.056	−28.1 ± 0.68
	20.2	61.5	0.94 ± 0.07	0.16 ± 0.04	0.931	0.049	−28.2 ± 1.21
	45.7	90.4	1.75 ± 0.05	0.00 ± 0.03	0.950	0.064	−21.2 ± 0.89
	70.9	100	4.57 ± 0.05	0.07 ± 0.01	0.975	0.046	−21.4 ± 0.06
	94.8	66.7	1.02 ± 0.08	0.00 ± 0.06	0.913	0.061	−19.6 ± 0.89
0.5 Hz|18 mm|A1	0	17.6	0.55 ± 0.14	0.00 ± 0.10	0.892	0.021	−29.8 ± 1.14
	27.1	19.8	1.77 ± 0.05	0.21 ± 0.02	0.971	0.012	−24.8 ± 0.89
	51.0	29.8	1.32 ± 0.03	0.03 ± 0.02	0.989	0.010	−26.0 ± 1.36
	74.9	22.0	1.47 ± 0.05	0.00 ± 0.03	0.965	0.012	−28.8 ± 1.30
	94.2	16.8	1.96 ± 0.06	0.00 ± 0.03	0.948	0.011	−28.9 ± 2.05
0.5 Hz|18 mm|A2	0	25.9	0.68 ± 0.06	0.66 ± 0.04	0.952	0.021	−30.1 ± 1.16
	20.0	36.2	0.69 ± 0.05	0.08 ± 0.04	0.978	0.017	−27.3 ± 1.00
	47.6	43.0	1.86 ± 0.06	0.40 ± 0.01	0.941	0.029	−23.5 ± 1.05
	71.2	38.1	1.37 ± 0.04	0.00 ± 0.03	0.980	0.018	−19.5 ± 1.30
	93.8	30.2	1.50 ± 0.06	0.00 ± 0.04	0.948	0.020	−26.4 ± 2.08
2.5 Hz|6 mm|A1	0	ND	ND	ND	ND	ND	−26.1 ± 1.04
	21.8	22.8	0.82 ± 0.05	0.39 ± 0.02	0.968	0.011	−24.1 ± 0.80
	46.9	26.4	0.40 ± 0.06	0.00 ± 0.04	0.984	0.009	−30.6 ± 1.33
	70.7	23.0	1.56 ± 0.03	0.00 ± 0.02	0.985	0.008	−31.8 ± 1.17
	94.6	23.8	1.89 ± 0.04	0.00 ± 0.02	0.971	0.010	−30.8 ± 1.03
2.5 Hz|6 mm|A2	0	36.7	1.11 ± 0.10	0.38 ± 0.04	0.851	0.038	−29.2 ± 1.13
	21.7	31.7	1.22 ± 0.05	0.29 ± 0.02	0.961	0.025	−25.4 ± 1.07
	48.7	50.0	1.11 ± 0.05	0.15 ± 0.03	0.967	0.025	−21.9 ± 1.78
	70.1	50.2	1.45 ± 0.03	0.00 ± 0.02	0.986	0.017	−21.6 ± 0.80
	94.5	33.5	1.56 ± 0.03	0.00 ± 0.02	0.984	0.012	−27.2 ± 1.77
2.5 Hz|18 mm|A1	0	13.2	*	*	*	*	−27.2 ± 0.95
	23.8	27.6	0.51 ± 0.06	0.44 ± 0.06	0.970	0.014	−23.4 ± 1.05
	48.2	56.3	1.21 ± 0.03	0.18 ± 0.02	0.988	0.018	−21.8 ± 1.04
	72.3	44.2	1.71 ± 0.03	0.34 ± 0.01	0.984	0.019	−27.1 ± 0.84
	92.1	34.5	1.06 ± 0.05	0.00 ± 0.04	0.973	0.016	−29.6 ± 0.29
2.5 Hz|18 mm|A2	0	36.3	*	*	*	*	−21.6 ± 0.31
	22.9	34.5	0.97 ± 0.04	0.33 ± 0.01	0.978	0.014	−22.1 ± 1.57
	45.0	53.6	1.47 ± 0.02	0.00 ± 0.01	0.989	0.017	−18.3 ± 0.75
	74.8	63.5	1.97 ± 0.06	0.13 ± 0.02	0.999	0.006	−25.9 ± 2.46
	95.0	41.8	0.67 ± 0.24	0.00 ± 0.21	0.857	0.042	−29.2 ± 1.91

*k*—sedimentation constant; ND—not determined; RMSE—root mean squared error; R^2^—coefficient of determination; t_c_—cultivation time; *λ*—lag time; values are presented as the mean ± standard deviation; ζ—zeta potential. * Fit did not converge.

**Table 4:** Statistically significant regression parameters (*p* < 0.05) and corresponding β values obtained from the multiple linear regression (MLR) analysis.

Parameters	MLR					
	*β_0_*	*β_1_*	*β_2_*	*β_3_*	*β_4_*	*β_5_*
*y*_1_	1.45 ± 0.23					
*y*_2_	0.48 ± 0.07					0.13 ± 0.07
*y*_3_	1.08 ± 0.37	0.47 ± 0.38		0.60 ± 0.39	0.42 ± 0.39	−0.93 ± 0.40
*y*_4_	0.32 ± 0.06			0.06 ± 0.06		−0.10 ± 0.06
*y*_5_	0.07 ± 0.01	−0.02 ± 0.01		0.02 ± 0.01		−0.02 ± 0.01

*β*_0_: independent parameter; *β*_1_: cultivation time; *β*_2_: oscillation amplitude; *β*_3_: oscillation frequency; *β*_4_: sedimentation kinetic constant on day 0 k_to_; *β*_5_: zeta potential. *y*_1_: sedimentation kinetic constant; *y*_2_: sedimentation efficiency; *y*_3_: daily biomass productivity; *y*_4_: nitrogen removal rate; *y*_5_: phosphorous removal rate.

**Table 5:** Summary of the principal component analysis (PCA) results based on the collected experimental data, with component selection guided by the Kaiser criterion.

	PC1	PC2	PC3
*x*_1_	0.024	0.016	**−0.949**
*x*_2_	**0.879**	0.035	−0.088
*x*_3_	0.004	**0.803**	0.201
*x*_4_	**−0.883**	0.127	−0.070
*x*_5_	0.096	**−0.769**	0.276
Eigenvalue	1.63	1.22	1.00
Variance (%)	32.52	24.44	19.97
Cumulative variance (%)	32.52	56.96	76.93

*x*_1_: cultivation time; *x*_2_: amplitude; *x*_3_: frequency; *x*_4_: sedimentation kinetic constant on day 0 k_to_; *x*_5_: zeta potential. Values in bold indicate the variables that mostly influenced the corresponding principal component.

**Table 6:** Statistically significant regression parameters (*p* < 0.05) and corresponding β values obtained from the principal component regression (PCR) analysis.

Parameters	PCR			
	*β*_0_	*β*_1_	*β*_2_	*β*_3_
*y*_1_	1.45 ± 0.21			−0.28 ± 0.22
*y*_2_	0.48 ± 0.06		−0.11 ± 0.05	
*y*_3_	1.08 ± 0.37	−0.53 ± 0.23	0.95 ± 0.30	
*y*_4_	0.32 ± 0.06		0.08 ± 0.05	0.07 ± 0.06
*y*_5_	0.07 ± 0.01		0.02 ± 0.01	0.02 ± 0.01

*β*_0_: independent parameter; *β*_1_: PC1; *β*_2_: PC2; *β*_3_: PC3; *y*_1_: sedimentation kinetic constant; *y*_2_: sedimentation efficiency; *y*_3_: daily biomass productivity; *y*_4_: nitrogen removal rate; *y*_5_: phosphorous removal rate.

## Data Availability

The original contributions presented in this study are included in the article/Supplementary Material. Further inquiries can be directed to the corresponding author(s).

## Supplementary Materials

The following supporting information can be downloaded at: https://www.mdpi.com/article/10.3390/biotech15020036/s1, Table S1: Specific growth rate (µ, d^−1^) and sedimentation efficiency after 2 h on the third day of cultivation (SE, %) under different oscillation frequency and amplitude conditions; Table S2: Power density estimates for the tested oscillatory conditions; Table S3: Calibration curve data for the analysis of biomass, nitrogen, and phosphorus concentrations; Figure S1: Contour plots of specific growth rate (a) and sedimentation efficiency (b) as a function of oscillation frequency and amplitude; Figure S2: Relative OD ration at 680 nm, from the beginning to the end of the experiments (t0 to t4), fitted according to the modified Gompertz model, measured over time for the following conditions: (A) A1 0.5 Hz|6 mm; (B) A2 0.5 Hz|6 mm; (C) A1 0.5 Hz|18 mm; (D) A2 0.5 Hz|18 mm; (E) A1 2.5 Hz|6 mm; (F) A2 2.5 Hz|6 mm; (G) A1 2.5 Hz|18 mm; (H) A2 2.5 Hz|18 mm.
