# Supplementary material for: Evaluation of Oscillatory Flow Conditions for Microalgal CO2 Capture and Biomass Sedimentation Kinetics: Experimental and Mathematical Approach
Source: BioTech (Basel). 2026 May 23;15(2):36. doi: 10.3390/biotech15020036 (PMC13297455; doi:10.3390/biotech15020036)
Supplement: Supplementary file 1 [file biotech-15-00036-s001.zip › biotech-4224697-supplementary.pdf]

Supplementary Material

Evaluation of oscillatory flow conditions for microalgal CO_2 capture and biomass sedimentation kinetics: experimental and mathematical approach

Inês S. Almeida, Eva M. Salgado*, António M.A. Ferreira, José C.M. Pires*

LEPABE, ALiCE, Faculty of Engineering, University of Porto, Rua Dr. Roberto Frias, 4200-465 Porto, Portugal.

* Corresponding authors

Telephone: +351 22 041 3685

E-mail addresses: up201606419@edu.fe.up.pt (E.M.S.), jcpires@fe.up.pt (J.C.M.P.).

Section I – Previous Experiments

The current section presents the results from previous experiments conducted by the research team using the same reactor under similar cultivation conditions. A Design of Experiments (DoE) was conducted to test oscillation frequencies of 0.5 and 1.5 Hz and amplitudes of 6 and 12 mm. The results are presented in Table S1 and Figure S1.

Table S1 Specific growth rate (μ, d^{-1}) and sedimentation efficiency after 2 hours on the third day of cultivation (SE, %) under different oscillation frequency and amplitude conditions

Assay	Frequency (Hz)	Amplitude (mm)	μ (d^{-1})	SE (%)
1	0.5	6	1.31	32.00
2	0.5	6	1.12	29.79
3	1.5	12	2.45	48.54
4	0.5	12	1.03	41.86
5	1.5	6	1.65	41.16
6	0.5	12	1.97	25.59
7	1.5	6	1.80	34.56
8	1.5	12	2.00	21.97

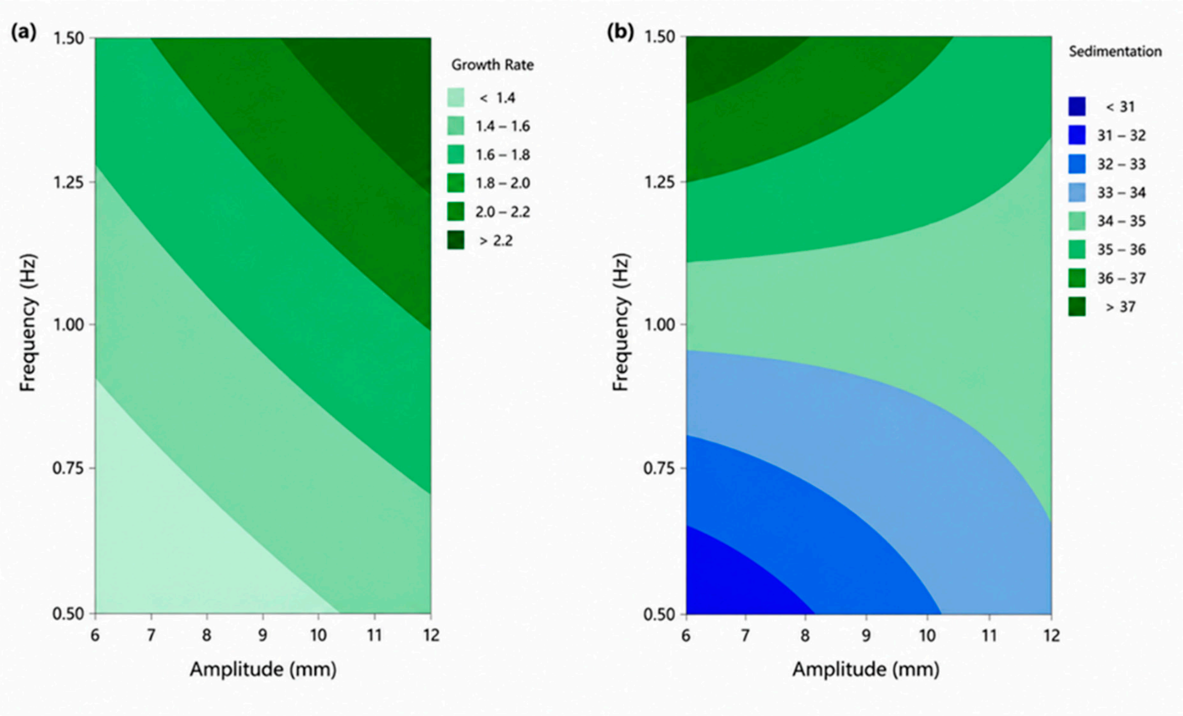

Figure S1 Contour plots of specific growth rate (a) and sedimentation efficiency (b) as a function of oscillation frequency and amplitude

Section II – Supplementary Figures and Tables

Table S2 Power density and energy estimates for the tested oscillatory conditions

f (Hz)	x_0 (mm)	Qg (cm^3 min^{-1})	Ug (m s^{-1})	Reo	$(P/V)_0$ (W m^{-3})	$(P/V)_B$ (W m^{-3})	P/V (W m^{-3})	Energy consumption (kWh m^{-3})
0.5	6	60	0.00455	380	1.24	44.47	46	4.42
0.5	18	60	0.00455	1140	33.44	44.47	78	7.49
2.5	6	60	0.00455	1900	154.83	44.47	199	19.10
2.5	18	60	0.00601	4957	4180.45	58.80	4239	406.94

Energy consumption calculated as: P/V × 96h/1000. f: oscillatory frequency; Qg: gas flow rate; Reo: oscillatory Reynolds number; Ug: superficial gas velocity; x0: oscillation amplitude; P/V_0 : power density from oscillatory flow; P/V_B : power density from bubble rise; P/V: total power density. Fixed parameters: $\alpha = 0.189$, N = 15.87, D = 18.4 mm, Dh = 16.72 mm, $\varrho = 997.05$ kg m^{-3}, $\mu = 9.10 \times 10^{-4}$ kg m^{-1} s^{-1}.

Table S3 Calibration curve data for analysis of biomass, nitrogen, and phosphorus concentrations

x	y	Linearity range	a ± s_a	b ± s_b	R	n_s
X (mg_{DW} L^{-1})	$Abs_{680\,nm}$	28 – 563 mg_{DW} L^{-1}	$(3.00 \pm 0.07) \times 10^{-3}$	$(7.50 \pm 2.30) \times 10^{-2}$	0.9936	10
NO_3-N (mg_N L^{-1})	$Abs_{220\,nm}$	1-49 mg_N L^{-1}	$(1.30 \pm 0.01) \times 10^{-2}$	$(1.51 \pm 0.49) \times 10^{-2}$	0.9994	8
PO_4-P (mg_P L^{-1})	$Abs_{820\,nm}$	0.11 – 4.55 mg_P L^{-1}	$(1.84 \pm 0.02) \times 10^{-1}$	$(4.00 \pm 3.00) \times 10^{-3}$	0.9996	7

a: slope; Abs: absorbance; b: intercept; DW: dry weight; NO_3-N: nitrate-nitrogen; n_s: number of standards; PO_4-P: phosphate-phosphorus; R: correlation coefficient; s_a: standard deviation of the slope; s_b: standard deviation of the intercept.

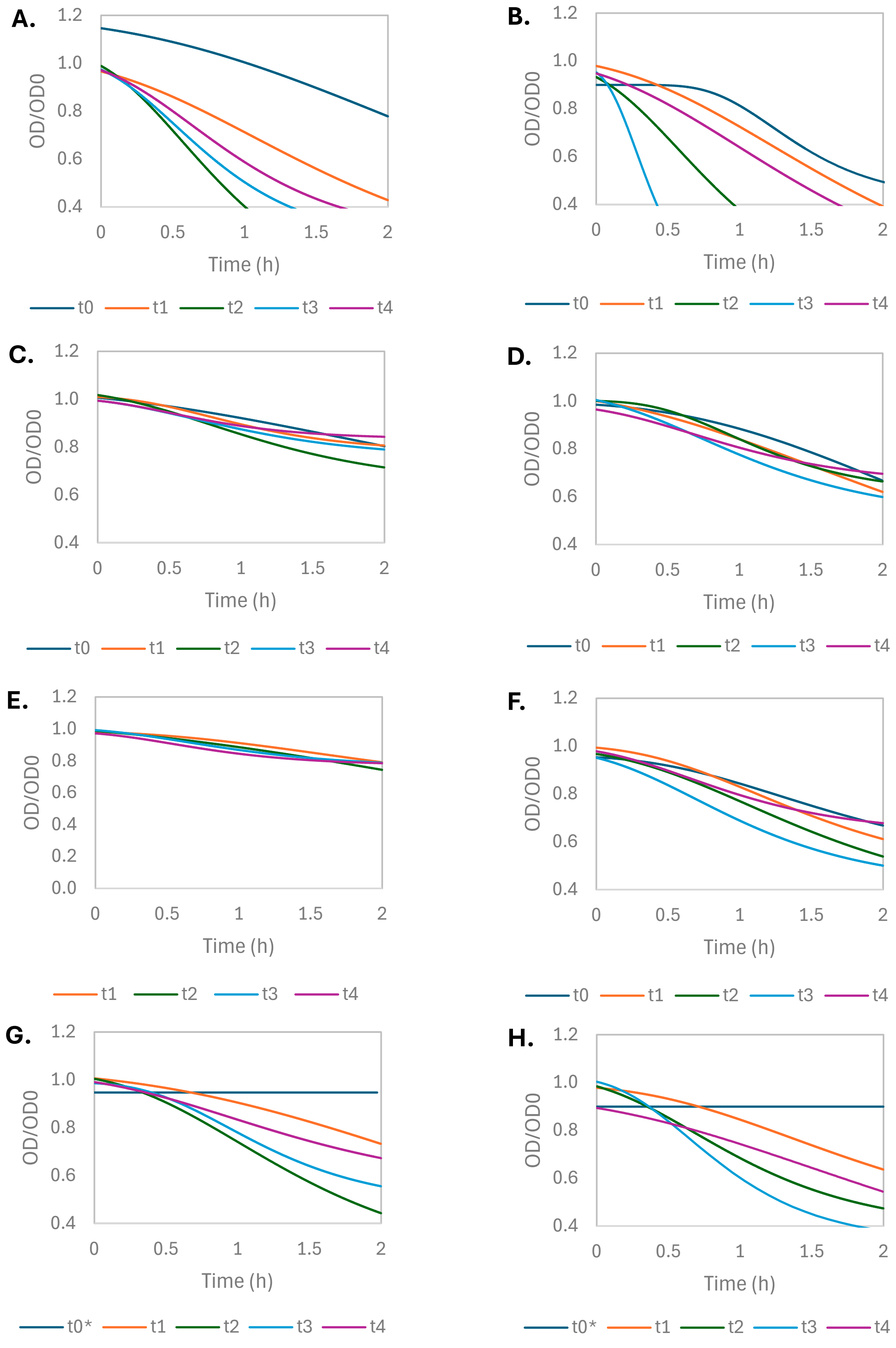

Figure S2 Relative OD ration at 680 nm, from the beginning to the end of the experiments (t0 to t4), fitted according to the modified Gompertz model, measured over time for the following conditions: (A) A1 0.5 Hz | 6 mm; (B) A2 0.5 Hz | 6 mm; (C) A1 0.5 Hz | 18 mm; (D) A2 0.5 Hz | 18 mm; (E) A1 2.5 Hz | 6 mm; (F) A2 2.5 Hz | 6 mm; (G) A1 2.5 Hz | 18 mm; (H) A2 2.5 Hz | 18 mm.
